# Supplementary material for: Performances of Functional and Anatomic Imaging Modalities in Succinate Dehydrogenase A-Related Metastatic Pheochromocytoma and Paraganglioma
Source: Cancers (Basel). 2022 Aug 11;14(16):3886. doi: 10.3390/cancers14163886 (PMC9406057; doi:10.3390/cancers14163886)
Supplement: Supplementary file 1 [file cancers-14-03886-s001.zip › cancers-1837805-supplementary.pdf]

Supplementary Material

Figure S1. Contingency Tables for all 11 patients comparing lesions identified on 68Ga-DOTATATE versus 18F-FDG, 18F-FDOPA, and CT/MRI

=0.0853	68Ga-DOTATATE			
18F-FDG		+	-	total
	+	203	30	233
	-	46	2	48
	total	249	32	281

<0.0001	68Ga-DOTATATE			
18F-FDOPA		+	-	total
	+	25	14	39
	-	55	8	63
	total	80	22	102

<0.0001	68Ga-DOTATATE			
CT/MRI		+	-	total
	+	135	103	238
	-	24	8	32
	total	159	111	270

Figure S2. Contingency Tables for 7 patients where all four imaging modalities were conducted comparing the lesions identified on 68Ga-DOTATATE versus 18F-FDG, 18F-FDOPA, and CT/MRI

=1.00	68Ga-DOTATATE			
18F-FDG		+	-	total
	+	61	20	81
	-	19	2	21
	total	80	22	102

<0.0001	68Ga-DOTATATE			
18F-FDOPA		+	-	total
	+	25	14	39
	-	55	8	63
	total	80	22	102

<0.0010	68Ga-DOTATATE			
CT/MRI		+	-	total
	+	41	14	55
	-	39	8	47
	total	80	22	102
